# Cell-autonomous role of Presenilin in age-dependent survival of cortical interneurons

**DOI:** 10.1186/s13024-020-00419-y

**Published:** 2020-12-10

**Authors:** Jongkyun Kang, Jie Shen

**Affiliations:** 1 Department of Neurology, Brigham and Women’s Hospital, Boston, MA 02115 USA; 2 Program in Neuroscience, Harvard Medical School, Boston, MA 02115 USA

**Keywords:** Alzheimer’s disease, Conditional knockout mouse, Cerebral cortex, GABAergic, Interneurons, Excitatory neurons, Apoptosis, Neuronal death, Astrogliosis, Microgliosis

## Abstract

**Background:**

Mutations in the *PSEN1* and *PSEN2* genes are the major cause of familial Alzheimer’s disease. Previous studies demonstrated that Presenilin (PS), the catalytic subunit of γ-secretase, is required for survival of excitatory neurons in the cerebral cortex during aging. However, the role of PS in inhibitory interneurons had not been explored.

**Methods:**

To determine PS function in GABAergic neurons, we generated inhibitory neuron-specific *PS* conditional double knockout (IN-*PS* cDKO) mice, in which PS is selectively inactivated by Cre recombinase expressed under the control of the endogenous *GAD2* promoter. We then performed behavioral, biochemical, and histological analyses to evaluate the consequences of selective PS inactivation in inhibitory neurons.

**Results:**

IN-*PS* cDKO mice exhibit earlier mortality and lower body weight despite normal food intake and basal activity. Western analysis of protein lysates from various brain sub-regions of IN-*PS* cDKO mice showed significant reduction of PS1 levels and dramatic accumulation of γ-secretase substrates. Interestingly, IN-*PS* cDKO mice develop age-dependent loss of GABAergic neurons, as shown by normal number of GAD67-immunoreactive interneurons in the cerebral cortex at 2–3 months of age but reduced number of cortical interneurons at 9 months. Moreover, age-dependent reduction of Parvalbumin- and Somatostatin-immunoreactive interneurons is more pronounced in the neocortex and hippocampus of IN-*PS* cDKO mice. Consistent with these findings, the number of apoptotic cells is elevated in the cerebral cortex of IN-*PS* cDKO mice, and the enhanced apoptosis is due to dramatic increases of apoptotic interneurons, whereas the number of apoptotic excitatory neurons is unaffected. Furthermore, progressive loss of interneurons in the cerebral cortex of IN-*PS* cDKO mice is accompanied with astrogliosis and microgliosis.

**Conclusion:**

Our results together support a cell-autonomous role of PS in the survival of cortical interneurons during aging. Together with earlier studies, these findings demonstrate a universal, essential requirement of PS in the survival of both excitatory and inhibitory neurons during aging.

**Supplementary Information:**

The online version contains supplementary material available at 10.1186/s13024-020-00419-y.

## Background

Alzheimer’s disease (AD) is the most common neurodegenerative disorder afflicting ~ 50 million people worldwide. Dominantly inherited mutations in the *Presenilin* genes (*PSEN1* and *PSEN2*) account for ~ 90% of mutations identified in familial Alzheimer’s disease (FAD), highlighting their importance in the pathogenesis [1]. Presenilin-1 (PS1) and PS2 are highly expressed in neurons throughout development and in adulthood [2–4], and PS is the catalytic subunit of the γ-secretase complex [5]. Genetic studies in mice showed that during development PS regulates neurogenesis and cell-fate decisions in the brain [6–8] as well as tumorigenesis in the skin [9]. In the adult cerebral cortex, PS/γ-secretase plays an essential role in excitatory neurons; selective inactivation of PS or another essential component of the γ-secretase complex, Nicastrin, in excitatory neurons of postnatal forebrain results in memory impairment, deficits in synaptic plasticity, and age-dependent neurodegeneration including gliosis [10–19].

The cerebral cortex is composed of glutamatergic excitatory projection neurons and local GABAergic inhibitory interneurons, and inhibitory neurons regulate the overall balance of excitatory and inhibitory neuronal activities via inhibitory neurotransmitter, γ-aminobutyric acid (GABA). Interneurons represent 10–20% of the neuronal populations in the cerebral cortex, and they are a highly heterogenous group of neurons with distinct morphological, electrophysiological, and neurochemical characteristics [20–22]. The largest group of cortical interneurons is calcium binding protein Parvalbumin (PV)-expressing neurons, which represent ~ 40% total interneurons. PV-expressing interneurons provide somatic inhibition to pyramidal neurons and display fast-spiking activity. A second class of interneurons expresses the neuropeptide somatostatin (SST), and it represents ~ 30% of total interneurons in the cortex. SST-expressing interneurons provide distal inhibition and display more gradual, delayed response to stimulation. A third group of cortical interneurons generally express the serotonin receptor 5HT3aR, but this interneuron group is very diverse with varying morphologies and releasing neuromodulators [23, 24]. It has been reported that the number of inhibitory neurons, especially PV- or SST-expressing interneurons, is reduced in various sub-regions of postmortem AD brains, such as the entorhinal cortex and the hippocampus [25–31]. In addition, dysfunction of GABAergic neurotransmission and imbalance of the neural network have emerged as one of the potential mechanisms of cognitive dysfunction in AD [32, 33]. Despite the importance of inhibitory neurons in AD pathogenesis, the role of PS in inhibitory neurons had not been studied.

To address this important question, we generated inhibitory neuron-specific *PS* conditional double knockout mice (IN-*PS* cDKO), in which PS is selectively inactivated in GABAergic inhibitory neurons using the *GAD2-IRES-Cre* driver [34]. IN-*PS* cDKO mice are fertile but exhibit earlier mortality. Importantly, IN-*PS* cDKO mice exhibit age-dependent loss of interneurons and increases of apoptosis as well as astrogliosis and microgliosis in the cerebral cortex, demonstrating an essential protective role of PS in the survival of cortical interneurons during aging.

## Methods

### Generation of inhibitory neuron-specific *PS* cDKO mice

All mice were housed in humidity- and temperature-controlled rooms maintained on a 12:12 h light: dark cycle, and were given standard rodent chow and water. To generate inhibitory neuron-specific *PS* cDKO (IN-*PS* cDKO) mice, we first crossed homozygous floxed *PS1* (*fPS1/fPS1*); *PS2*−/− mice [13] with *GAD2-IRES-Cre* knockin (KI) mice (The Jackson Laboratory, 010802, RRID: IMSR_JAX:010802) [34] to obtain *fPS1/+; PS2*+/−*; GAD2-IRES-Cre/+* mice, which were then bred with *fPS1/fPS1; PS2*−/− mice to generate *fPS1/fPS1; PS2*−/−*; GAD2-IRES-Cre/+* (IN-*PS* cDKO) mice. IN-*PS* cDKO and littermate control mice used in the study were obtained from breeding *fPS1/fPS1; PS2*−/− mice together with *fPS1/fPS1; PS2*−/−*; GAD2-IRES-Cre/+* or *fPS1/+; PS2*−/−*; GAD2-IRES-Cre/+* mice. Both IN-*PS* cDKO male and female mice are viable and fertile with normal size litters (7.34 ± 0.23 mice per litter, total 279 pups from 38 litters). Genotyping was performed at postnatal days 10–12, and the progeny of intercrosses of IN-*PS* cDKO and control mice were found at the expected Mendelian ratio (F_1_ = 1.294, *p* = 0.26, Chi-square; Control: *N* = 130, cDKO: *N* = 149).

Both male and female mice were used in each experiment. IN-*PS* cDKO and littermate control mice were maintained in the C57BL/6 J 129 hybrid genetic background. The experimenter of all behavioral and quantitative histological analyses was blind to the genotype of the mice.

All procedures were approved by the IACUC committees of Harvard Medical School and Brigham and Women’s Hospital, and conform to the USDA Animal Welfare Act, PHS Policy on Humane Care and Use of Laboratory Animals, the “ILAR Guide for the Care and Use of Laboratory Animals” and other applicable laws and regulations.

### PCR genotyping

Tail genomic DNA was extracted at postnatal days 10–12. The primers used in the PCR to differentiate the wild-type, floxed or deleted *PS1* allele are P140 (5′-TCAACTCCTCCAGAGTCAGG, forward primer in *PS1* intron 1), P158 (5′-TGCCCCCTCTCCATTTTCTC, reverse primer in *PS1* intron 1), and P139 (5′-GGTTTCCCTCCATCTTGGTTG, reverse primer in *PS1* intron 3). The 216 bp PCR product (from P140-P158) represents the wild-type *PS1* allele, whereas the 262 bp fragment represents the floxed *PS1* allele. The 372 bp PCR product (from P140-P139) represents the deleted *PS1* allele. The primers used in the PCR to differentiate the wild-type or deleted *PS2* allele are P162 (5′-CATCTACACGCCCTTCACGG, forward primer in *PS2* exon 5), P163 (5′-CACACAGAGAGGCTCAGGATC, reverse primer in *PS2* intron 5), and P164 (5′-AAGGGCCAGCTCATTCCTCC, forward primer in the *PGK* sequences of the *PGK-Neo* selection cassette). The 581 bp PCR product (from P162-P163) represents the wild-type *PS2* allele, whereas the 326 bp PCR product (from P163-P164) represents the deleted *PS2* allele. The primers used in the PCR to differentiate the wild-type or the *GAD2-IRES-Cre* KI allele are JKM028 (5′-CTTCTTCCGCATGGTCATCT, forward primer in the coding sequences of *GAD2* exon 16), JKM029 (5′-CACCCCACTGGTTTTGATTT, reverse primer in the 3′ UTR of *GAD2* exon 16), and JKM030 (5′-AAAGCAATAGCATCACAAATTTCA, reverse primer in the SV40 polyA signal sequences of *IRES-Cre*, inserted immediately following the stop codon of the *GAD2* gene in exon 16). The 250 bp PCR product (from JKM028-JKM029) represents the wild-type *GAD2* allele, whereas the 352 bp fragment (from JKM028-JKM030) represents the C*re* KI allele.

### Daily food intake analysis

IN-*PS* cDKO mice (*N* = 8, 5 male and 3 female) and littermate controls (*N* = 8, 5 male and 3 female) at 2 months of age were separated into single-housed cages. Food intake of individual mice was measured daily for 7 days, and an average daily food intake per mouse was calculated by averaging the quantity of food consumed over 7 days.

### Open field test

IN-*PS* cDKO mice (*N* = 11, 9 male and 2 female) and littermate controls (*N* = 10, 5 male and 5 female) at 2 months of age were used for the analysis. Individual mice were handled daily for 5 days before testing. During the test, individual mice were placed in 42 × 42 cm acrylic animal cages for 15 min during which their horizontal and vertical movements were monitored by three arrays of 16 infrared light beam sensors (AccuScan Instruments) connected to a computer that recorded their position every millisecond. AccuScan VersaMax software was then used to calculate, in both the horizontal plane and along the vertical axis, the total number of movements, distance traveled, time spent moving, and the total number of infrared beam breaks for each mouse.

### Western analysis

Each brain sub-region was dissected from the harvested brain. Fresh tissues were homogenized in an ice-cold stringent RIPA buffer [50 mM Tris-Cl (pH 7.6), 150 mM NaCl, 0.5 mM EDTA, 1% NP40, 0.5% sodium deoxycholate, 0.1% SDS, 1 mM PMSF supplement with protease inhibitor cocktail and phosphatase inhibitor cocktail (Sigma)], followed by sonication. Homogenates were centrifuged at 14,000 rpm for 20 min at 4 °C to separate supernatants (RIPA buffer-soluble fraction). Equal amounts (10–40 μg per lane) of total proteins from each preparation were loaded and separated on NuPAGE gels (Invitrogen), and transferred to nitrocellulose membranes. The membranes were blocked in 5% skim milk/TBS for 1 h, and incubated at 4 °C overnight with specific primary antibodies. The primary antibodies used were mouse anit-PS1 (MAB5232, MilliporeSigma, 1/1000, RRID: AB_95175), rabbit anti-APP-Y188 (ab32136, Abcam, 1/2000, RRID: AB_2289606), rabbit anti-MAP 2 (#4542, Cell Signaling, 1/1000, RRID: AB_776174), rabbit anti-SYP (#5431, Cell Signaling, 1/1000, RRID: AB_10698743), rabbit anti-SNAP25 (ab108990, abcam, 1/2000, RRID: AB_10888111), rabbit anti-PSD95 (#2507, Cell Signaling, 1/1000, RRID: AB_1264242), mouse anti-GFAP (G6171, 1/2000, Sigma-Aldrich, RRID: AB_1840893), or mouse anti-β-actin (#3700, Cell Signaling, 1/4000, RRID: AB_2242334). Membranes were then incubated with secondary antibodies, which was either goat anti-rabbit IRdye680 (926–68,071, LI-COR Bioscience, 1/20000, RRID: AB_10956166), goat anti-mouse IRdye680 (926–68,072, LI-COR Bioscience, 1/20000, RRID: AB_10953628), or goat anti-mouse IRdye800 (926–32,210, LI-COR Bioscience, 1/20000, RRID: AB_621842). Signals were quantified using the Odyssey Infrared Imaging System (LI-COR Biosciences).

### Histological analysis

Mice were anesthetized with ketamine (100 mg/kg) + xylazine (10 mg/kg) + acepromazine (3 mg/kg), and transcardially perfused with phosphate-buffered saline solution (PBS, pH 7.4) containing 0.25 g/L heparin (Sigma) and 5 g/L procaine (Sigma). Brains were post-fixed in 4% formaldehyde in PBS (Electron Microscopy Sciences) at 4 °C overnight and then processed for paraffin embedding following standard procedures. Serial sagittal sections (10 μm) were obtained using Leica RM2235. For Nissl staining, paraffin sagittal sections were deparaffinized, dehydrated, and stained with 0.5% cresyl violet (Sigma-Aldrich). Immunohistochemical analysis was performed as previously described [35]. Briefly, paraffin sagittal sections were deparaffinized, alcohol-dehydrated, then subjected to permeabilization with a solution containing 0.1% Triton X-100, 0.1% sodium citrate in PBS, except those for cleaved-caspase3 immunostaining, which were performed antigen retrieval by microwaving for 10 min in 10 mM sodium citrate buffer, pH 6.0. Endogenous peroxidase activity was quenched by incubating in 0.3% H_2_O_2_ in methanol. Sections were then blocked with a PBS solution containing 5% normal goat serum (Vector Laboratories) for 1 h at room temperature. After blocking, sections were incubated with primary antibodies overnight at 4 °C. The primary antibodies used were mouse anti-GAD67 (MAB5406, MilliporeSigma, 1/1000, RRID: AB_2278725), mouse anti-PV (P3088, MilliporeSigma, 1/1000, RRID: AB_744329), rat anti-SST (MAB354, MilliporeSigma, 1/300, RRID: AB_2255365), rabbit anti-cleaved caspases-3 (#9661, Cell Signaling Technology, 1/150, RRID: AB_2341188), mouse anti-GFAP (G6171, Sigma-Aldrich, 1/500, RRID: AB_1840893), or rabbit anti-Iba1 (#019–19,741, Wako, 1/300, RRID: AB_839504). Sections were then incubated for 1 h with biotinylated secondary antibodies at room temperature, followed by 1 h incubation with Vectastain Elite ABC reagent, and then developed using chromogenic DAB substrate (Vector Laboratories, RRID: AB_2336827).

For the TUNEL assay, deparaffinized and rehydrated brain sections were subjected to permeabilization with a solution containing 0.1% Triton X-100, 0.1% sodium citrate in PBS for 8 min. Sections were then blocked with a solution containing 5% normal goat serum (Vector Laboratories) for 1 h at room temperature, followed by the protocol of the manufacturer of the In Situ Cell Death Detection kit (Roche). The slides were then washed using PBS three times. Images were captured and analyzed using an Olympus IX51 fluorescent microscope.

For the immunofluorescence staining, deparaffinized and rehydrated brain sections were subjected to permeabilization with a solution containing 0.1% Triton X-100, 0.1% sodium citrate in PBS, and antigen retrieval was performed by microwaving for 10 min in 10 mM sodium citrate buffer, pH 6.0. Sections were then blocked with a solution containing 5% normal goat serum (Vector Laboratories) for 1 h. After blocking, sections were incubated with primary antibodies overnight at 4 °C. The primary antibodies used were mouse anti-GAD67 (MAB5406, MilliporeSigma, 1/500, RRID: AB_2278725), mouse anti-CaMKIIα (MA1–048, Thermo Fisher Scientific, 1/200, RRID: AB_325403), or rabbit anti-cleaved caspases-3 (#9661, Cell Signaling Technology, 1/150, RRID: AB_2341188). Sections were then incubated for 1 h with fluorophore conjugated secondary antibodies, Alexa Fluor 488 goat anti-rabbit IgG (A-11034, Thermo Fisher Scientific, 1/250, RRID: AB_2576217) and Alexa Fluor 555 goat anti-mouse IgG (A-21424, Thermo Fisher Scientific, 1/250, RRID: AB_141780) at room temperature. Fluorescence images were taken and analyzed by FV1000 confocal microscope system (Olympus).

### Stereological quantification

Stereological quantification was performed as previously described using the BioQuant image analysis software that was connected to the Olympus BX51 microscope with a CCD camera [35]. Specifically, the volume of the neocortex or hippocampus was quantified using Nissl stained serial sections (every 40^th^ sagittal sections, spaced 0.4 mm apart; total 6–7 sections per hemisphere). The entire area of the neocortex or hippocampus was analyzed, and the data were calculated to reflect the volume of both hemispheres (*N* ≥ 6 mice per genotype per age group).

The number of GAD67-immunoreactive interneurons was quantified using GAD67 stained serial sections (every 40^th^ sagittal sections, spaced 0.4 mm apart; total 6–7 sections per hemisphere). An unbiased fractionator and optical dissector method [35] were used to count GAD67-immunoreactive cells in the neocortical area. Approximately forty optical dissectors of the 250 × 250 μm sampling box were used for the quantification. The number of neurons was counted with an indicator of immunopositive cells in the soma through the 20X objective lens from all the picked slides. In the hippocampus, the total number of GAD67-immunoreactive cells was calculated by multiplying 40 by the total number of immunopositive cells in the hippocampus of one hemisphere in all 6–7 sections. Finally, average number of neurons per hemisphere was calculated per genotype (*N* ≥ 6 mice per genotype per age group).

Similarly, the number of PV- or SST-immunoreactive inhibitory neurons was quantified using PV- or SST-stained serial sections (every 40^th^ sagittal sections, spaced 0.4 mm apart; total 6–7 sections per hemisphere). The total number of PV- or SST-immunoreactive cells in the neocortex or hippocampus was calculated by multiplying 40 with the total number of immunoreactive cells in each brain sub-region of one hemisphere in all 6–7 sections. Finally, average number of neurons per hemisphere was calculated per genotype (*N* ≥ 6 mice per genotype per age group).

The number of active caspase-3-immunoreactivity cells or TUNEL+ cells in the cerebral cortex was determined using serial sections (every 30^th^ sagittal sections, spaced 0.3 mm apart; 7–9 sections per hemisphere), and the number of positive cells was counted. The total number of active caspase-3-immunoreactivity or TUNEL+ cells in the cerebral cortex of the hemisphere was calculated by multiplying 30 with the total number of active caspase-3-immunopositive or TUNEL+ cells in each brain sub-region of one hemisphere in all 7–9 sections. Finally, average immunopositive cells or TUNEL+ cells per hemisphere was calculated per genotype (*N* ≥ 6 mice per genotype per age group).

The quantification of apoptotic cells labeled with active caspase-3-immunoreactivity and GAD67- or CaMKIIα-immunoreactivity was performed using serial sections (every 30^th^ sagittal sections, spaced 0.3 mm apart; 5 sections per hemisphere). Single or double immunolabeled cells were counted across the entire cerebral cortex area under FV1000 confocal microscope system (Olympus), and the number of positive cells per section was averaged per genotype (*N* ≥ 7 mice per genotype).

The GFAP-immunoreactive areas were analyzed using GFAP stained serial sections (every 40^th^ sagittal sections, spaced 0.4 mm apart; 6–7 sections per hemisphere). The GFAP-immunoreactive area and the total area of the neocortex or hippocampus, were measured under 4X objective, and the percentage of GFAP-immunoreactive area was calculated as GFAP+ area/total area. Finally, average GFAP+ area/total area % per hemisphere was calculated per genotype (*N* ≥ 6 mice per genotype per age group).

The Iba1-immuonreactive cells were analyzed using Iba1 stained serial sections (every 40^th^ sagittal sections, spaced 0.4 mm apart; 6–7 sections per hemisphere). The number of Iba1+ cells was counted using the unbiased fractionator and optical dissector methods and the BioQuant image analysis software. Optical dissectors of the 100 × 100 μm sampling box were used for the quantification in the neocortex, and 250 × 250 μm sampling box were used for the quantification in the hippocampus. The Iba1+ signal was calculated as the Iba1+ cell number/total area (*N* ≥ 7 mice per genotype). Values are reported as mean ± SEM. The coefficient of error from the stereological counting technique was < 0.10.

### Experimental design and statistical analysis

Data acquisition and quantification were performed in a genotype blind manner except for the molecular analysis. All statistical analysis was performed using Prism 8 (GraphPad software) or Excel (Microsoft). All data are presented as mean ± SEM. The exact sample size, the number of mice or neurons, of each experiment is indicated in the figure or the legend.

Statistical analyses were conducted using two-tailed Student’s *t*-test for the comparison of a given variable in two genotypes or two-way ANOVA followed by Bonferroni’s post hoc comparisons for the comparison of more than two conditions. All statistical comparisons were performed on data from ≥6 biologically independent samples and replicated on different experimental days. Significance is shown as *p < 0.05, **p < 0.01, ***p < 0.001, ****p < 0.0001, and not significantly different values are often not noted.

## Results

### Generation and molecular validation of IN-*PS* cDKO mice

To investigate the normal physiological role of PS in inhibitory interneurons, we generated IN-*PS* cDKO mice, in which PS inactivation is restricted to inhibitory interneurons by the *GAD2-IRES-Cre* driver [34], resulting in Cre-mediated deletion of *PS1* exons 2–3 [10, 13, 36]. Similar to the generation of excitatory neuron-specific *PS* cDKO (EX-*PS* cDKO) mice using an excitatory neuron specific driver, *Camk2a-Cre* [10, 13], mice homozygous for the floxed *PS1* (*fPS1*) and *PS2*-null alleles (*fPS1/fPS1; PS2−/−*) were crossed with *GAD2-IRES-Cre* KI mice to obtain *fPS1/+; PS2+/−; GAD2-IRES-Cre/+* mice, which were then bred with *fPS1/fPS1; PS2−/−* to generate IN-*PS* cDKO (*fPS1/fPS1; PS2−/−; GAD2-IRES-Cre/+*). IN-*PS* cDKO mice were further bred with *fPS1/fPS1; PS2−/−* mice to obtain IN-*PS* cDKO mice and littermate controls (*fPS1/fPS1; PS2−/−*) for phenotypic characterization. IN-*PS* cDKO mice were obtained at the expected Mendelian ratio at postnatal days 12–14, indicating that they survive normally during the embryonic and early postnatal development.

**Fig. 1:**
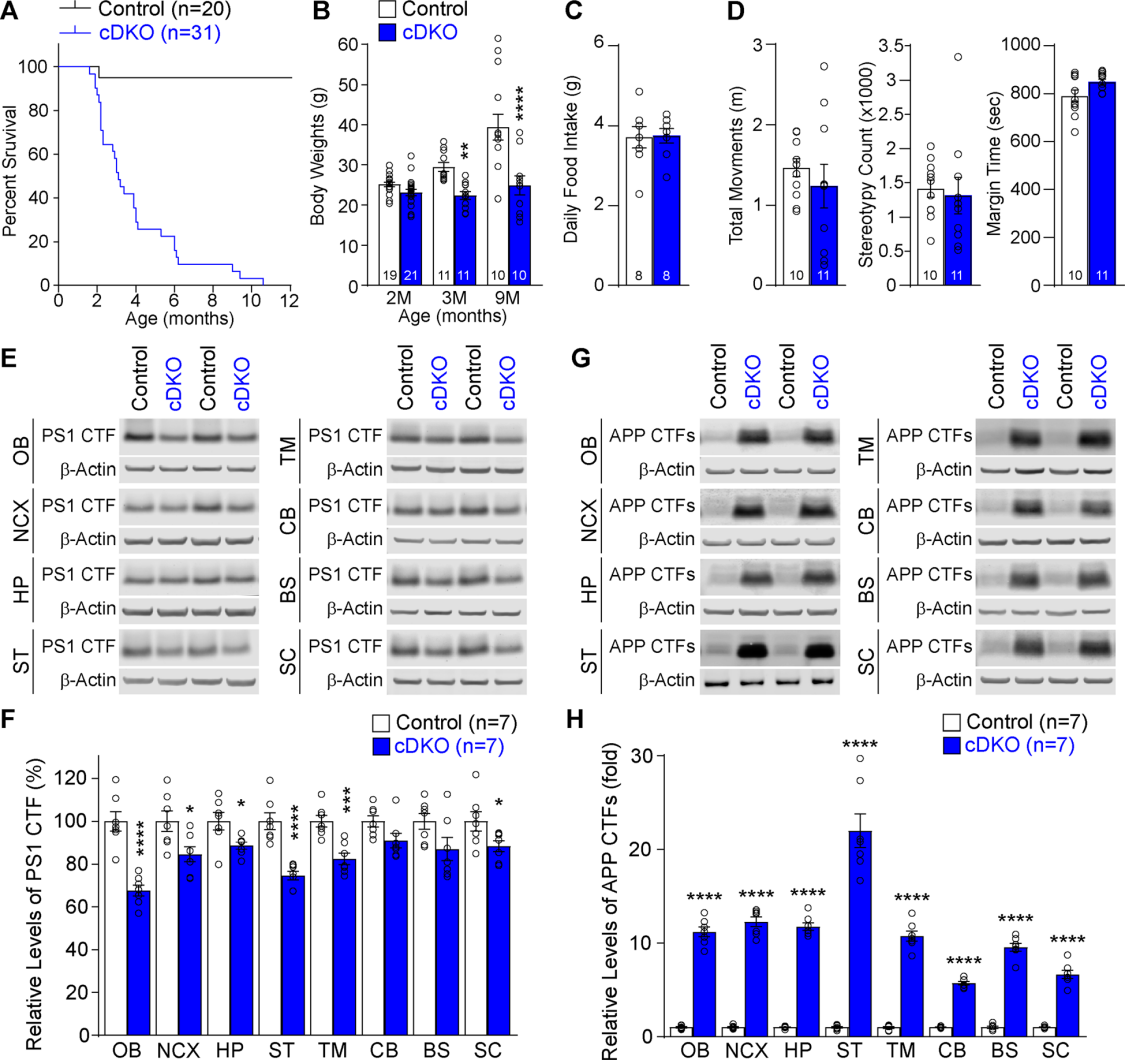
Reduction of PS1 levels and γ-secretase activity in the brain of IN-*PS* cDKO mice. **a** IN-*PS* cDKO mice exhibit dramatically earlier mortality as shown by the survival graph of IN*-PS* cDKO mice and littermate controls. Survival curves are plotted using the Kaplan-Meier method. **b** IN*-PS* cDKO mice fail to gain weight during aging, compared to littermate controls (F_2, 76_ = 9.69, *p* = 0.0002; 2 M: *p* = 0.68, 3 M: *p* = 0.0061, 9 M: *p* < 0.0001, two-way ANOVA with Bonferroni’s post hoc comparisons). **c** Daily food intake of controls and IN-*PS* cDKO mice on chew diet at 2 months of age. Bar graph shows no significant difference in daily food intake amount between controls and IN-*PS* cDKO mice (*p* = 0.91, Student’s *t*-test). Daily food intake was calculated by averaging 7 days of food consumption per mouse. **d** During the open field test, IN-*PS* cDKO and control mice display similar total movements (*p* = 0.43, Student’s *t*-test), stereotypy count (*p* = 0.73), and the time spent of margin (*p* = 0.06) at 2 months of age. **e** Western blotting of total protein lysates from the dissected olfactory bulb (OB), neocortex (NCX), hippocampus (HP), striatum (ST), thalamus and midbrain (TM), cerebellum (CB), brain stem (BS), and spinal cord (SC) of control and IN*-PS* cDKO mice at 2 months of age. β-Actin was used as internal loading control. **f** Quantification analysis shows significant reduction of the PS1 CTF in the OB (*p* < 0.0001, Student’s *t*-test), NCX (*p* = 0.0230), HP (*p* = 0.0233), ST (*p* < 0.0001), TM (*p* = 0.0005), and SC (*p* = 0.0445) of IN*-PS* cDKO mice relative to littermate controls at the age of 2 months. The level of the PS1 CTF in the CB (*p* = 0.0577) and BS (*p* = 0.0704) of IN*-PS* cDKO mice is not statistically different from that of control mice. The value of the PS1 CTF in controls is set as 100%. **g** Western blotting of total protein lysates from the dissected olfactory bulb (OB), neocortex (NCX), hippocampus (HP), striatum (ST), thalamus and midbrain (TM), cerebellum (CB), brain stem (BS), and spinal cord (SC) of the controls and IN*-PS* cDKO mice at 2 months of age shows dramatic increases of APP CTFs in IN-*PS* cDKO mice. β-Actin was used as internal loading control. **h** Quantification analysis shows accumulation of APP CTFs in various brain sub-regions of IN-*PS* cDKO relative to controls at the age of 2 months (*p* < 0.0001, Student’s *t*-test). The value of APP CTFs in controls is set as 1. All data represent mean ± SEM. *p < 0.05, **p < 0.01, ***p < 0.001, ****p < 0.0001. The value in the column indicates the number of mice used in each experiment

Interestingly, IN-*PS* cDKO mice show early mortality; they start to die after 2 months of age, and few live beyond 11 months of age (Fig. 1a). While IN-*PS* cDKO mice exhibit normal body weight at 2 months of age, they show significantly lower body weight, relative to controls, at the ages of 3 and 9 months (Fig. 1b). However, the brain weight of IN-*PS* cDKO mice is normal relative to controls at all ages examined (e.g. at 9 months, Control: 492.2 ± 10.8 mg, cDKO: 490.3 ± 15.6 mg, *p* = 0.92, Student’s *t*-test). To determine whether the lower body weight and the earlier mortality of IN-*PS* cDKO mice are due to poor feeding, we measured their daily food consumption, and found no difference between IN-*PS* cDKO mice and controls (Fig. 1c). We further evaluated IN-*PS* cDKO and control mice in the open field test, and found no genotypic difference in total movements (*p* = 0.43, Student’s *t*-test), stereotypy counts (*p* = 0.73), and time spent in the margin or the center (*p* = 0.06) of the open field arena (Fig. 1d).

It was previously shown that the *GAD2-IRES-Cre* KI allele drives Cre-mediated recombination in > 90% of GAD67-immunoreactive cortical interneurons [34]. We therefore anticipated that *GAD2-IRES-Cre* would similarly delete the floxed *PS1* exons 2–3 in GABAergic interneurons of IN-*PS* cDKO mice. Due to the lack of specific PS1 antibodies that can be used for co-immunohistochemical analysis of PS1 and GAD67 in IN-*PS* cDKO brains, we performed Western analysis using protein lysates from dissected brain sub-regions from IN-*PS* cDKO mice and littermate controls at the age of 2 months to verify the reduction of PS1 expression in the IN-*PS* cDKO brain. We found that levels of the PS1 C-terminal fragment (CTF) are significantly decreased in the olfactory bulb (*p* < 0.0001, Student’s *t*-test), neocortex (*p* = 0.0230), hippocampus (*p* = 0.0233), striatum (*p* < 0.0001), thalamus/midbrain (*p* = 0.0005), and spinal cord (*p* = 0.0445), relative to littermate controls (Fig. 1e, f). The varying degree of partial reductions of the PS1 CTF is consistent with the varying percentage of GABAergic neurons present in the brain sub-regions, as lysates contain proteins not only from interneurons but also from excitatory neurons and glial populations, in which Cre is not expressed thus PS1 expression unaffected.

We previously reported that in the cerebral cortex of EX-*PS* cDKO mice, the CTFs of the amyloid precursor protein (APP), which are physiological substrates of γ-secretase, accumulate to very high levels (30-fold) relative to control mice at 2 months of age, due to the complete inactivation of PS/γ-secretase in cortical excitatory neurons [10]. We therefore similarly measured levels of APP CTFs in various sub-regions of IN-*PS* cDKO brains at 2 months of age. Western analysis indeed showed dramatic accumulation of APP CTFs to varying degrees in all brain sub-regions examined with ~ 12-fold accumulation in the neocortex and hippocampus of IN-*PS* cDKO mice (Fig. 1g, h). These results are consistent with the lower percentage (10–20%) of interneurons, relative to excitatory neurons, in the cerebral cortex. Due to the low levels of APP CTFs in the control brain and the very high levels of APP CTFs in the IN-*PS* cDKO brain, which makes accurate estimation more challenging, we performed serial dilutions (1:2, 1:3, 1:5, 1:10, 1:20, 1:30) of lysates from each of the brain sub-regions of IN-*PS* cDKO mice followed by Western blotting (Supplemental Figure 1). We found similar fold accumulation of APP CTFs in various brain sub-regions of IN-*PS* cDKO mice. These results confirmed inactivation of PS and loss of γ-secretase activity in IN-*PS* cDKO brains.

### Normal generation of cortical interneurons in IN-*PS* cDKO mice

**Fig. 2:**
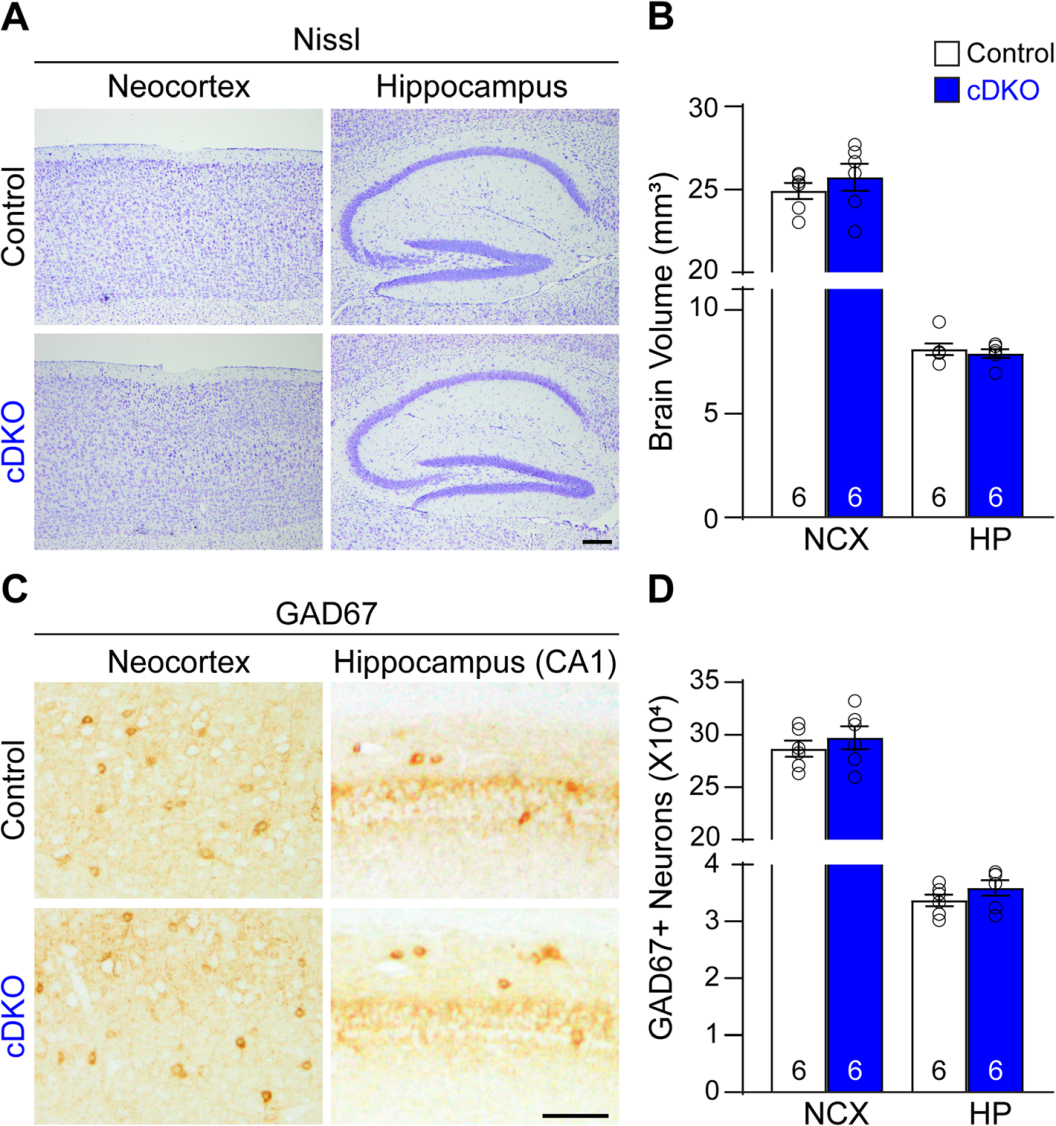
Normal cortical interneurons in IN-*PS* cDKO mice at 2 months of age. **a** Nissl staining of comparable sagittal sections of IN-*PS* cDKO mice and littermate control brains at 2 months of age shows normal gross morphology in the neocortex (NCX) and hippocampus (HP) of IN-*PS* cDKO mice. **b** Stereological quantification shows similar volume of the NCX (*p* = 0.41, Student’s *t*-test) and HP (*p* = 0.55) between IN-*PS* cDKO and control mice. **c** GAD67 immunostaining of comparable sagittal sections in the neocortex and hippocampal area CA1 of IN-*PS* cDKO and littermate control brains at 2 months of age. **d** Stereological quantification shows similar numbers of GAD67-immunoreactive neurons in the NCX (*p* = 0.45, Student’s *t*-test) and HP (*p* = 0.23) between IN-*PS* cDKO and control mice. Scale bar: 100 μm. All data are presented as the mean ± SEM. The value in the column indicates the number of mice used in each experiment

Cre recombinase expressed from the *GAD2-IRES-Cre* KI allele is under the control of the *GAD2* promoter, which begins its expression approximately at embryonic day 10.5 [37]. We therefore performed histological analysis of IN-*PS* cDKO and control brains at 2 months of age to determine whether generation and specification of GABAergic neurons is affected in IN-*PS* cDKO mice. Since the cerebral cortex is the major brain sub-region affected by AD pathogenesis, we focused our phenotypic analysis on the neocortex and the hippocampus. Nissl staining revealed no gross abnormality in IN-*PS* cDKO brains (Fig. 2a). Stereological quantification showed similar volume of the neocortex (Control: 24.91 ± 0.49 mm^3^; cDKO: 25.73 ± 0.81 mm^3^, *p* = 0.41, Student’s *t*-test) and the hippocampus (Control: 8.11 ± 0.28 mm^3^; cDKO: 7.89 ± 0.20 mm^3^, *p* = 0.55) between IN-*PS* cDKO mice and littermate controls (Fig. 2b). Inhibitory interneurons labeled by GAD67 immunoreactivity appear normal in the neocortex and hippocampus of IN-*PS* cDKO brains (Fig. 2c). Stereological quantification showed similar number of GAD67-immunopositive inhibitory interneurons in the neocortex (Control: 2.87 ± 0.08 × 10^5^; cDKO: 2.97 ± 0.11 × 10^5^, *p* = 0.45, Student’s *t*-test) and the hippocampus (Control: 3.37 ± 0.10 × 10^4^; cDKO: 3.59 ± 0.14 × 10^4^, *p* = 0.23) between IN-*PS* cDKO mice and littermate controls (Fig. 2d). These results indicate that selective inactivation of PS in the IN-*PS* cDKO embryonic brain by *GAD2-IRES-Cre* does not affect the generation and specification of interneurons in the cerebral cortex.

### Age-dependent loss of cortical interneurons in IN-*PS* cDKO mice

**Fig. 3:**
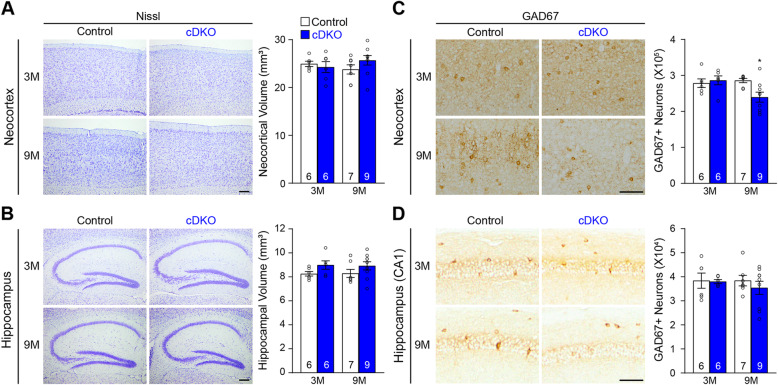
Progressive reduction of cortical interneurons in IN-*PS* cDKO mice. **a** *Left*: Nissl staining of comparable sagittal sections of IN-*PS* cDKO mice and littermate control brains at the ages of 3 and 9 months shows normal gross morphology in the neocortex of IN-*PS* cDKO mice. *Right*: Stereological quantification shows similar volume of the neocortex between IN-*PS* cDKO and control mice at 3 and 9 months of age (F_1, 24_ = 1.74, *p* = 0.20; 3 M: *p* > 0.99, 9 M: *p* = 0.31, two-way ANOVA with Bonferroni’s post hoc comparisons). **b** *Left*: Nissl staining of comparable sagittal sections of IN-*PS* cDKO mice and littermate control brains at the age of 3 and 9 months shows normal gross morphology in the hippocampus of IN-*PS* cDKO mice. *Right*: Stereological quantification shows similar volume of the hippocampus between IN-*PS* cDKO and control mice at 3 and 9 months of age (F_1, 24_ = 0.02, *p* = 0.88; 3 M: *p* = 0.31, 9 M: *p* = 0.32, two-way ANOVA with Bonferroni’s post hoc comparisons). **c** *Left*: GAD67 immunostaining of comparable sagittal sections in the neocortex of IN-*PS* cDKO and littermate control brains at 3 and 9 months of age. *Right*: Stereological quantification shows age-dependent decreases in the number of GAD67-immunoreactive interneurons in the neocortex of IN-*PS* cDKO mice compared to that in control mice (F_1, 24_ = 4.89, *p* = 0.0368; 3 M: Control 2.79 ± 0.12 × 10^5^, cDKO 2.86 ± 0.12 × 10^5^, *p* > 0.99, 9 M: Control 2.86 ± 0.06 × 10^5^, cDKO 2.40 ± 0.14 × 10^5^, *p* = 0.0167, two-way ANOVA with Bonferroni’s post hoc comparisons). **d** *Left*: GAD67 immunostaining of comparable sagittal sections in hippocampal area CA1 of IN-*PS* cDKO and littermate control brains at 3 and 9 months of age. *Right*: Stereological quantification shows no significant decrease in the total number of GAD67-immunoreactive interneurons in the entire hippocampus of IN-*PS* cDKO mice compared to that in control mice (F_1, 24_ = 0.26, *p* = 0.61; 3 M: Control 3.84 ± 0.32 × 10^4^, cDKO 3.80 ± 0.08 × 10^4^, *p* > 0.99; 9 M: Control 3.84 ± 0.22 × 10^4^, cDKO 3.54 ± 0.28 × 10^4^, *p* = 0.75, two-way ANOVA with Bonferroni’s post hoc comparisons). Scale bar: 100 μm. All data represent mean ± SEM. *p < 0.05. The value in the column indicates the number of mice used in each experiment

We further examined whether selective inactivation of PS in inhibitory interneuron affects their survival during aging. We performed histological analysis of IN-*PS* cDKO and control brains at the ages of 3 and 9 months. Nissl staining showed normal cortical morphology in IN-*PS* cDKO brains, and stereological quantification revealed unchanged volume of the neocortex and hippocampus in IN-*PS* cDKO mice at the age of 3 or 9 months (Fig. 3a, b). We also quantified the number of inhibitory interneurons in the cerebral cortex of IN-*PS* cDKO mice and littermate controls. Stereological quantification revealed that relative to control mice, the number of GAD67-immunoreactive interneurons is significantly reduced in the neocortex of IN-*PS* cDKO mice at 9 months (Control: 2.86 ± 0.06 × 10^5^, cDKO: 2.40 ± 0.14 × 10^5^, *p* = 0.0167, two-way ANOVA with Bonferroni’s post hoc comparisons) but not at 3 months (Fig. 3c). In the hippocampus, the number of GAD67-immunopositive neurons is not significantly altered in IN-*PS* cDKO mice at 3 or 9 months of age (Fig. 3d).

**Fig. 4:**
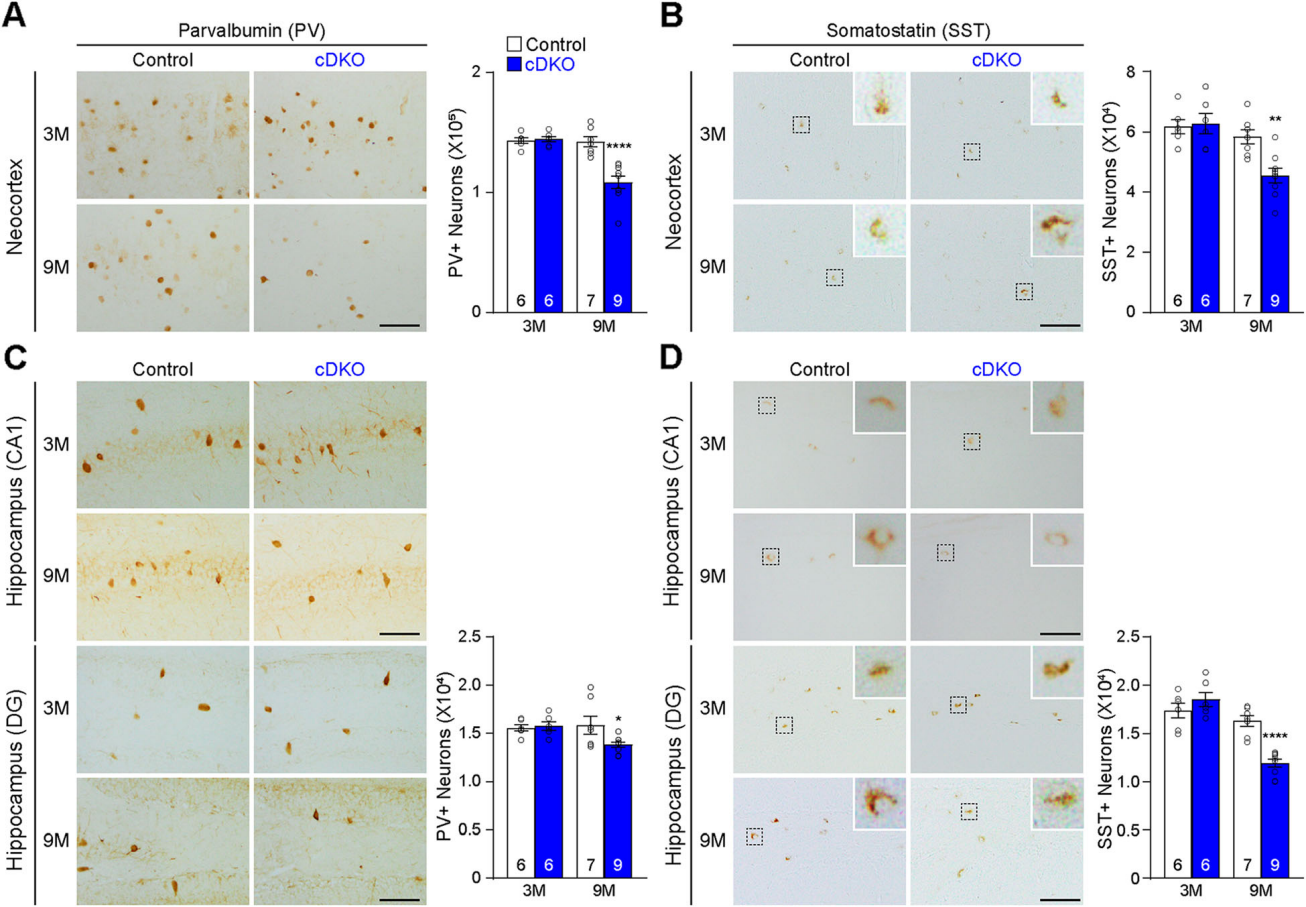
Progressive decreases of PV+ and SST+ interneurons in the cerebral cortex of IN-*PS* cDKO mice. **a** *Left*: PV immunostaining of comparable sagittal sections in the neocortex of IN-*PS* cDKO and littermate control brains at 3 and 9 months of age. *Right*: Stereological quantification shows age-dependent decreases in the number of PV-immunoreactive interneurons in the neocortex of IN-*PS* cDKO mice compared to that in control mice (F_1, 24_ = 16.20, *p* = 0.0005; 3 M: Control 1.43 ± 0.02 × 10^5^, cDKO 1.45 ± 0.02 × 10^5^, *p* > 0.99; 9 M: Control 1.43 ± 0.04 × 10^5^, cDKO 1.09 ± 0.05 × 10^5^, *p* < 0.0001, two-way ANOVA with Bonferroni’s post hoc comparisons). **b** *Left*: SST immunostaining of comparable sagittal sections in the neocortex of IN-*PS* cDKO and littermate control brains at 3 and 9 months of age. Inserts show higher power views of the boxed areas. *Right*: Stereological quantification shows age-dependent decreases in the number of SST-immunoreactive interneurons in the neocortex of IN-*PS* cDKO mice compared to that in control mice (F_1, 24_ = 6.61, *p* = 0.0168; 3 M: Control 6.17 ± 0.24 × 10^4^, cDKO: 6.28 ± 0.34 × 10^4^, *p* > 0.99; 9 M: Control 5.84 ± 0.24 × 10^4^, cDKO 4.55 ± 0.24 × 10^4^, *p* = 0.0027, two-way ANOVA with Bonferroni’s post hoc comparisons). **c** *Left*: PV immunostaining of comparable sagittal sections in hippocampus area CA1 (CA1) and the dentate gyrus (DG) of IN-*PS* cDKO and littermate control brains at 3 and 9 months of age. *Right*: Stereological quantification shows age-dependent decreases in the number of PV-immunoreactive interneurons in the entire hippocampus of IN-*PS* cDKO mice compared to that in control mice (F_1, 24_ = 3.84, *p* = 0.062; 3 M: Control 1.56 ± 0.03 × 10^4^, cDKO 1.58 ± 0.04 × 10^4^, *p* > 0.99; 9 M: Control 1.59 ± 0.10 × 10^4^, cDKO 1.38 ± 0.03 × 10^4^, *p* = 0.0244, two-way ANOVA with Bonferroni’s post hoc comparisons). **d** *Left*: SST immunostaining of comparable sagittal sections in hippocampus area CA1 (CA1) and the dentate gyrus (DG) of IN-*PS* cDKO and littermate control brains at 3 and 9 months of age. Inserts show higher power views of the boxed areas. *Right*: Stereological quantification shows age-dependent decreases in the number of SST-immunoreactive interneurons in the entire hippocampus of IN-*PS* cDKO mice compared to that in control mice (F_1, 24_ = 20.92, *p* = 0.0001; 3 M: Control 1.74 ± 0.08 × 10^4^, cDKO 1.85 ± 0.08 × 10^4^, *p* = 0.44; 9 M: Control 1.63 ± 0.06 × 10^4^, cDKO 1.19 ± 0.04 × 10^4^, *p* < 0.0001, two-way ANOVA with Bonferroni’s post hoc comparisons). Scale bar: 100 μm. All data represent mean ± SEM. *p < 0.05, **p < 0.01, ****p < 0.0001. The value in the column indicates the number of mice used in each experiment

We further examined two major sub-groups of inhibitory interneurons, Parvalbumin (PV)- or Somatostatin (SST)-expressing interneurons in the cerebral cortex of IN-*PS* cDKO mice. PV is a calcium binding protein, and is expressed in ~ 40% of GABAergic interneurons in the cerebral cortex, whereas SST is a neuropeptide expressed in ~ 30% of cortical interneurons [38–40]. To determine whether these sub-groups of interneurons are affected in IN-*PS* cDKO mice, we performed immunohistological analysis using antibodies specific for PV or SST, and quantified the number of PV+ or SST+ interneurons in the neocortex and hippocampus using a stereological method. We found that the number of PV-immunoreactive interneurons is significantly reduced in the neocortex of IN-*PS* cDKO mice at the age of 9 months (Control: 1.43 ± 0.04 × 10^5^, cDKO: 1.09 ± 0.05 × 10^5^, *p* < 0.0001, two-way ANOVA with Bonferroni’s post hoc comparisons) but not at 3 months (Fig. 4a). Similarly, the number of SST-immunoreactive interneurons is significantly reduced in the neocortex of IN-*PS* cDKO mice compared to controls at the age of 9 months (Control: 5.84 ± 0.24 × 10^4^, cDKO: 4.55 ± 0.24 × 10^4^, *p* = 0.0027, two-way ANOVA with Bonferroni’s post hoc comparisons) but not at 3 months (Fig. 4b). In the hippocampus, the number of PV-immunoreactive interneurons is also significantly decreased in IN-*PS* cDKO mice at the age of 9 months (Control: 1.59 ± 0.10 × 10^4^, cDKO: 1.38 ± 0.03 × 10^4^, *p* = 0.0244, two-way ANOVA with Bonferroni’s post hoc comparisons) but not at 3 months (Fig. 4c). Similarly, the number of SST-immunoreactive interneurons is markedly reduced in the hippocampus of IN-*PS* cDKO mice at the age of 9 months (Control: 1.63 ± 0.06 × 10^4^, cDKO: 1.19 ± 0.04 × 10^4^, *p* < 0.0001, two-way ANOVA with Bonferroni’s post hoc comparisons) but not at the age of 3 months (Fig. 4d). Thus, age-dependent loss of PV+ and SST+ cortical interneurons are more pronounced in IN-*PS* cDKO mice.

**Fig. 5:**
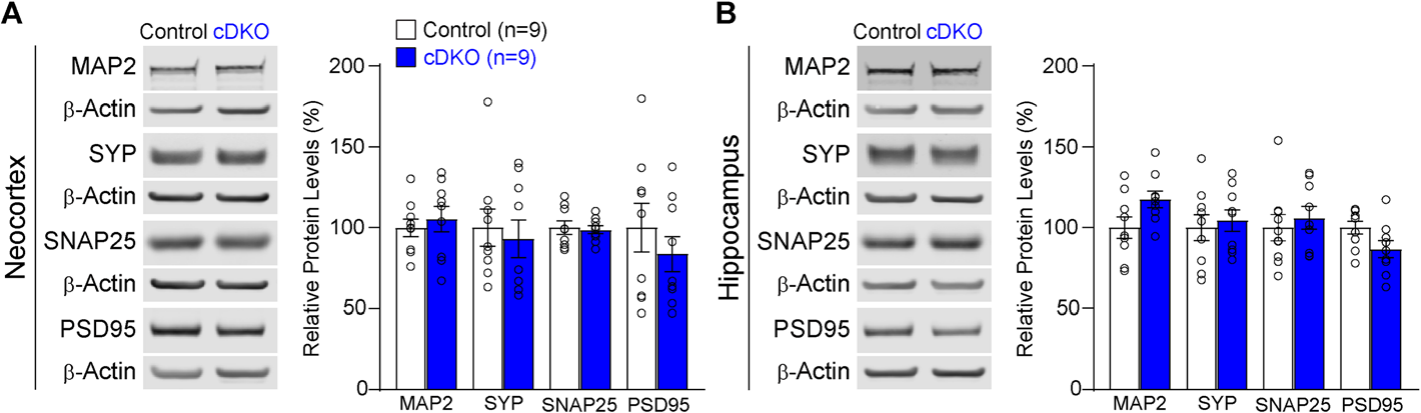
Normal levels of dendritic and synaptic makers in the cortex of IN-*PS* cDKO mice. **a** *Left*: Western blotting of neocortical lysates of IN-*PS* cDKO and controls at the age of 9 months. *Right*: Quantification analysis shows normal levels of dendritic and synaptic markers in the neocortex of IN-*PS* cDKO mice relative to controls (MAP2: *p* = 0.59, SYP: *p* = 0.68, SNAP25: *p* = 0.80, PSD95: *p* = 0.39, Student’s *t*-test). All values are normalized to β-Actin. **b** *Left*: Western blotting of hippocampal lysates of IN-*PS* cDKO and controls at the age of 9 months. *Right*: Quantification analysis shows normal levels of dendritic and synaptic markers in the hippocampus of IN-*PS* cDKO mice relative to controls (MAP2: *p* = 0.05, SYP: *p* = 0.68, SNAP25: *p* = 0.58, PSD95: *p* = 0.07, Student’s *t*-test). All values are normalized to β-Actin. All data represent mean ± SEM

We also examined whether levels of dendritic and synaptic markers are altered in the cerebral cortex of IN-*PS* cDKO mice at 9 months of age. Western analysis of dendritic marker MAP2, and synaptic markers, synaptophysin (SYP), synaptosomal nerve-associated protein 25 (SNAP25) and postsynaptic density protein 95 (PSD95) found no significant difference in their levels in the neocortex and hippocampus between IN-*PS* cDKO and control mice (Fig. 5a, b).

### Increases of apoptotic interneurons in the cerebral cortex of IN-*PS* cDKO mice

**Fig. 6:**
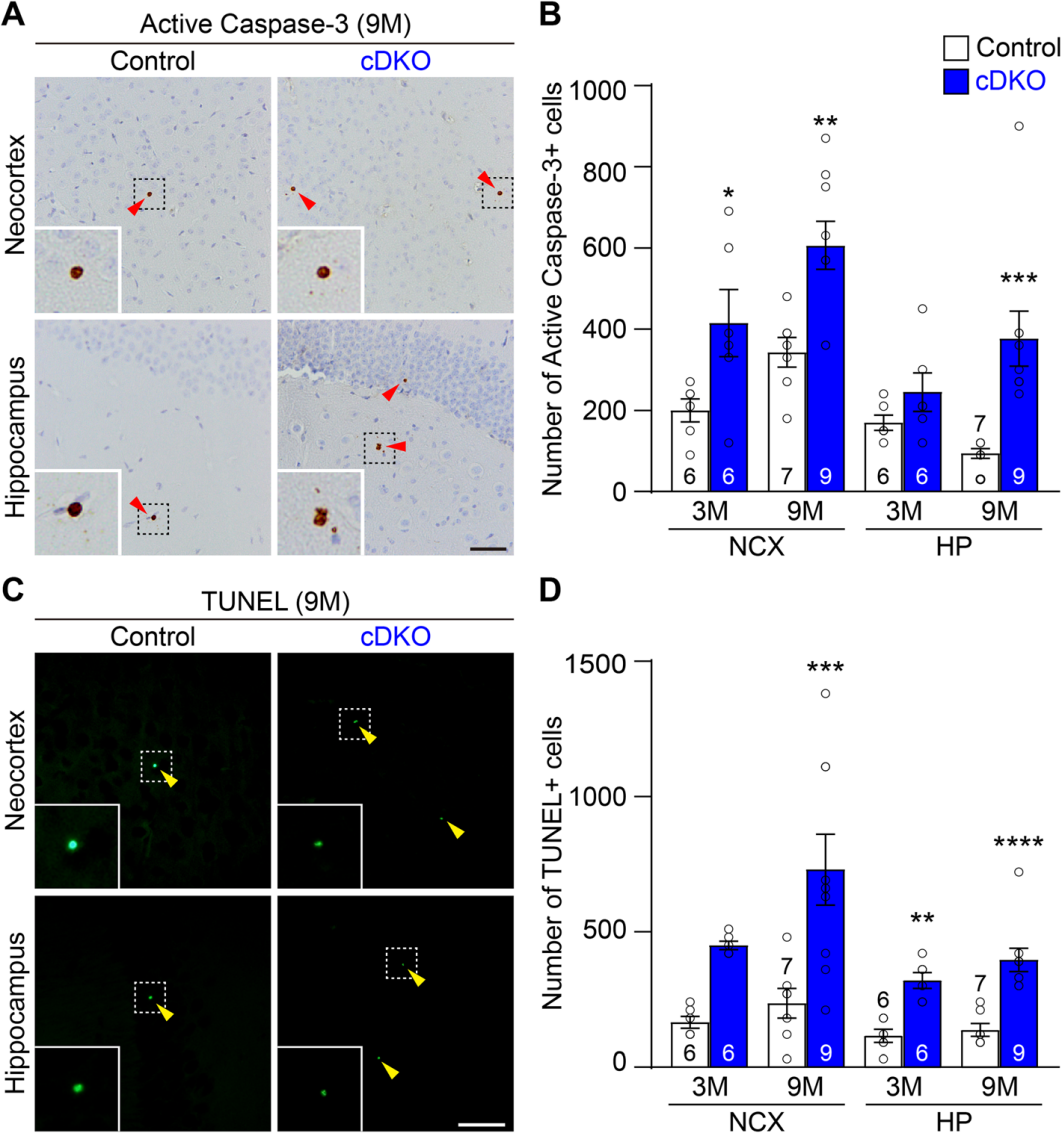
Increases of apoptotic cells in the cerebral cortex of IN-*PS* cDKO mice. **a** Representative images of active caspase-3-immunoreactive cells in the neocortex (NCX) and hippocampus (HP) of control and IN-*PS* cDKO mice at the age of 9 months. Active caspase-3-positive cells are shown in brown with red arrowheads. Inserts show higher power views of the boxed areas. The sections were counterstained with hematoxylin. Compared to controls, there are more cells positive of active caspase-3 in the NCX and HP of IN-*PS* cDKO mice. **b** Stereological quantification shows significant age-dependent increases in the number of active caspase-3-immunoreactive cells in the NCX (F_1, 24_ = 0.18, *p* = 0.67; 3 M: *p* = 0.0393, 9 M: *p* = 0.0036, two-way ANOVA with Bonferroni’s post hoc comparisons) and HP (F_1, 24_ = 4.22, *p* = 0.05; 3 M: *p* = 0.67, 9 M: *p* = 0.0006) of IN-*PS* cDKO mice compared to controls. **c** Representative images of TUNEL staining in the NCX and HP of littermate control and IN-*PS* cDKO mice at the age of 9 months. TUNEL+ cells are shown in green with yellow arrowheads. Inserts show higher power views of the boxed areas. More TUNEL+ cells are present in the NCX and HP of IN-*PS* cDKO mice compared to controls. **d** Stereological quantification shows significant age-dependent increases in the number of TUNEL+ cells in the NCX (F_1, 24_ = 1.30, *p* = 0.27; 3 M: *p* = 0.10, 9 M: *p* = 0.0008, two-way ANOVA with Bonferroni’s post hoc comparisons) and HP (F_1, 24_ = 0.61, *p* = 0.44; 3 M: *p* = 0.0014, 9 M: *p* < 0.0001) of IN-*PS* cDKO mice compared to controls. Scale bar: 100 μm. All data represent mean ± SEM. *p < 0.05, **p < 0.01, ***p < 0.001, ****p < 0.0001. The value in the column indicates the number of mice used in each experiment

The age-dependent loss of cortical interneurons in IN-*PS* cDKO mice prompted us to examine whether apoptosis is increased. We performed immunostaining using an antibody specific for the active form of caspase-3, a marker for apoptotic cells, in the brain of IN-*PS* cDKO mice and littermate controls at the ages of 3 and 9 months. Stereological quantification showed that the number of active caspase-3-immunopositive cells is significantly increased in the neocortex of IN-*PS* cDKO mice at the ages of 3 months (Control: 200.0 ± 27.6; cDKO: 415.0 ± 83.2, *p* = 0.0393, two-way ANOVA with Bonferroni’s post hoc multiple comparisons) and 9 months (Control: 342.9 ± 36.4; cDKO: 606.7 ± 58.5, *p* = 0.0036; Fig. 6a, b). Furthermore, the number of active casepase-3-immunopositive apoptotic cells is also significantly increased in the hippocampus of IN-*PS* cDKO mice at 9 months of age (Control: 94.3 ± 12.1; cDKO: 376.7 ± 68.0, *p* = 0.0006, two-way ANOVA with Bonferroni’s post hoc multiple comparisons; Fig. 6a, b).

We also performed the TUNEL assay, and found similar increases of TUNEL+ cells in the neocortex of IN-*PS* cDKO at the age of 9 months (Control: 235.7 ± 54.6, cDKO: 730.0 ± 130.8, *p* = 0.0008, two-way ANOVA with Bonferroni’s post hoc multiple comparisons; Fig. 6c, d). The number of TUNEL+ cells is also significantly increased in the hippocampus of IN-*PS* cDKO mice at 3 months of age (Control: 115.0 ± 23.8; cDKO: 320.0 ± 29.7; *p* = 0.0014, two-way ANOVA with Bonferroni’s post hoc multiple comparisons) and at 9 months of age (Control: 137.1 ± 23.5, cDKO: 396.7 ± 43.0, *p* < 0.0001; Fig. 6c, d).

**Fig. 7:**
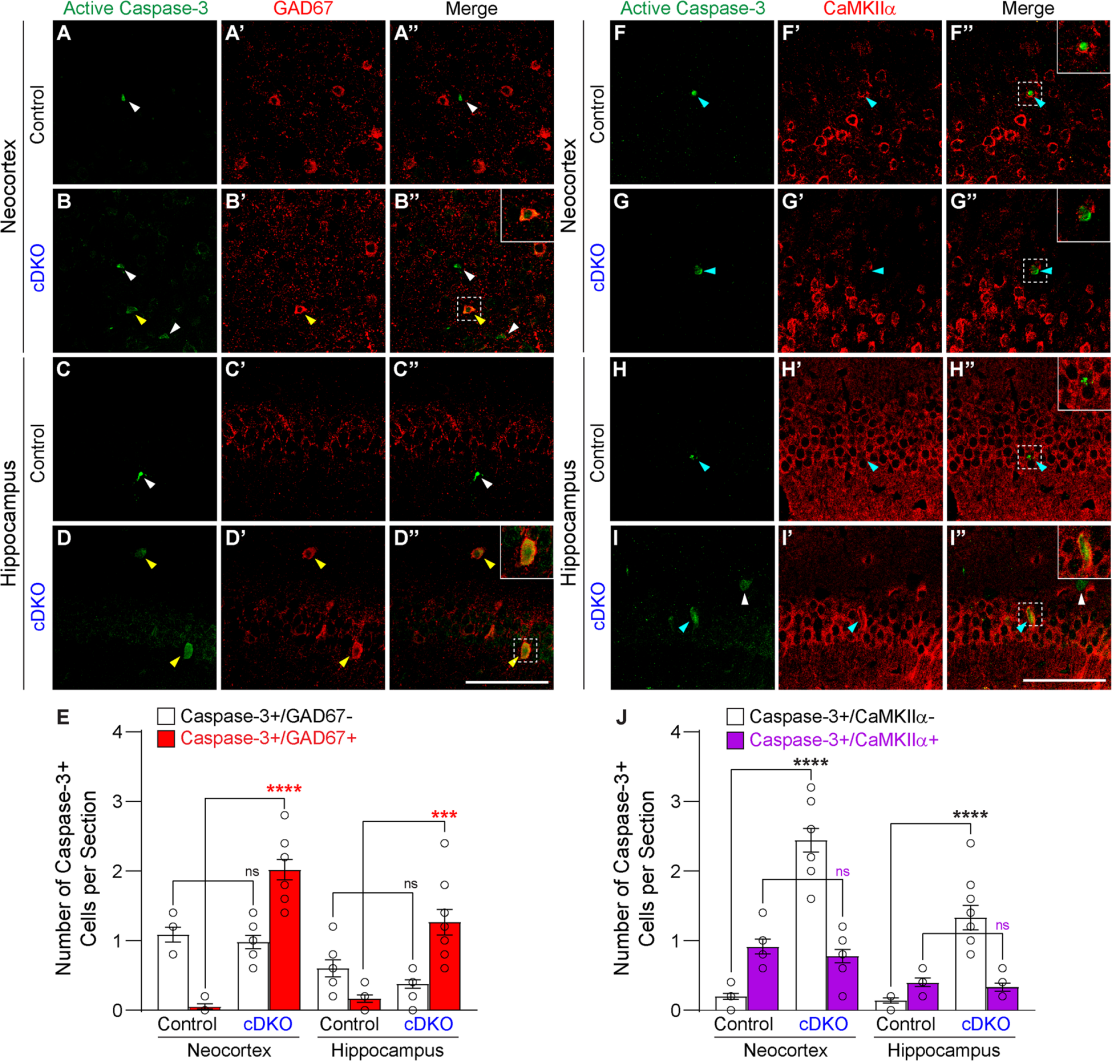
Dramatic increases of apoptotic interneurons in the cerebral cortex of IN-*PS* cDKO mice. **a-d** Immunohistochemical analysis shows co-localization of active caspase-3- and GAD67-immunoreactivity (yellow arrowheads) in the neocortex and hippocampus of IN-*PS* cDKO mice. The few apoptotic cells (active caspas-3+) present in the neocortex and hippocampus of the control brain tend to be GAD67- (white arrowheads). Inserts show higher power views of the boxed areas. **e** Quantification of apoptotic interneurons (active caspase-3+/GAD67+) and apoptotic non- interneurons (active caspase-3+/GAD67-) in the neocortex and hippocampus of control and IN-*PS* cDKO mice at the age of 9 months. IN-*PS* cDKO mice show dramatic increases of apoptotic interneurons (active caspase-3+/GAD67+) in the neocortex (*p* < 0.0001, Student’s *t*-test) and hippocampus (*p* = 0.0002), compared to controls. There is no significant difference in the number of apoptotic non-interneurons (active caspase-3+/GAD67-) in the neocortex (*p* = 0.47, Student’s *t*-test) and hippocampus (*p* = 0.11) between IN-*PS* cDKO and control mice. **f-i** The few cells immunoreactive for both active caspase-3 and CaMKIIα are shown (cyan arrowheads) in the neocortex and hippocampus of control and IN-*PS* cDKO brains, whereas most apoptotic cells are non-excitatory neurons (active caspase-3+/CaMKIIα-) present in IN-*PS* cDKO brains (white arrowheads). Inserts show higher power views of the boxed areas. **j** Quantification of apoptotic excitatory neurons (active caspase-3+/CaMKIIα+) and apoptotic non-excitatory neurons (active caspase-3+/CaMKIIα-) in the neocortex and hippocampus of control and IN-*PS* cDKO mice at the age of 9 months. While there are dramatic increases of apoptotic non-excitatory neurons in the neocortex (*p* < 0.0001, Student’s *t*-test) and hippocampus (*p* < 0.0001) of IN-*PS* cDKO mice, compared to controls, there is no significant difference in the number of apoptotic excitatory neurons in the neocortex (*p* = 0.36, Student’s *t*-test) and hippocampus (*p* = 0.45) of IN-*PS* cDKO mice, compared to controls. Scale bar: 100 μm. All data represent mean ± SEM. ns = no significance, ***p < 0.001, ****p < 0.0001

To determine the identity of the cells undergoing apoptosis in the neocortex and hippocampus of IN-*PS* cDKO mice, we performed co-immunohistological analysis using antibodies specific for active caspase-3 and CaMKIIα for excitatory neurons or GAD67 for inhibitory interneurons. We found dramatic increases of apoptotic interneurons (active caspase-3+/GAD67+) in the neocortex and hippocampus of IN-*PS* cDKO mice at 9 months of age, relative to controls (Fig. 7a-e). The number of non-interneuron apoptotic cells (active caspase-3+/GAD67-) in the neocortex and hippocampus is similar between control and IN-*PS* cDKO brains (Fig. 7e). Furthermore, the number of apoptotic excitatory neurons (active caspase-3+/CaMKIIα+) in the neocortex and hippocampus is similar between IN-*PS* cDKO and control brains, whereas the number of apoptotic non-excitatory neurons (active caspase-3+/CaMKIIα-) is much higher in the neocortex and hippocampus of IN-*PS* cDKO brains (Fig. 7f-j). These results together demonstrate that the increased apoptotic cells in IN-*PS* cDKO brains are interneurons rather than excitatory neurons.

### Progressive gliosis in the cerebral cortex of IN-*PS* cDKO mice

**Fig. 8:**
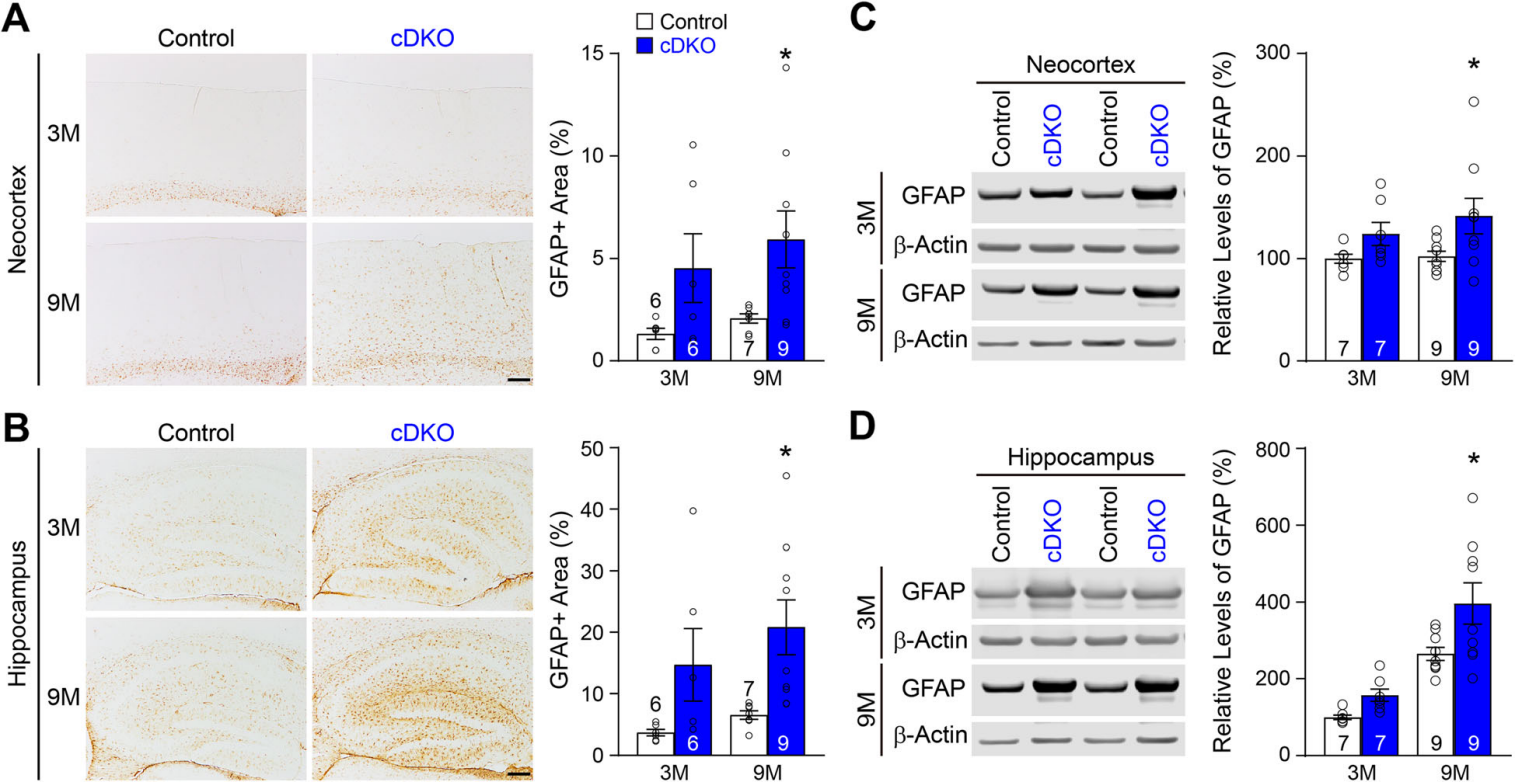
Progressive astrogliosis in the cerebral cortex of IN-*PS* cDKO mice. **a** *Left*: GFAP immunostaining of comparable sagittal sections in the neocortex of IN-*PS* cDKO and littermate control brains at 3 and 9 months of age. *Right*: Stereological quantification shows significant age-dependent increases in the percentage of GFAP-immunoreactive areas in the neocortex of IN-*PS* cDKO mice relative to control mice (F_1, 24_ = 0.08, *p* = 0.79; 3 M: Control 1.31 ± 0.27%, cDKO 4.53 ± 1.67%, *p* = 0.17; 9 M: Control 2.07 ± 0.22%, cDKO 5.93 ± 1.39%, *p* = 0.0399, two-way ANOVA with Bonferroni’s post hoc comparisons). **b** *Left*: GFAP immunostaining of comparable sagittal sections in the hippocampus of IN-*PS* cDKO and littermate control brains at 3 and 9 months of age. *Right*: Stereological quantification shows significant age-dependent increases in the percentage of GFAP-immunoreactive areas in the hippocampus of IN-*PS* cDKO mice relative to control mice (F_1, 24_ = 0.17, *p* = 0.68; 3 M: Control 3.71 ± 0.53%, cDKO 14.69 ± 5.90%, *p* = 0.15; 9 M: Control 6.57 ± 0.69%, cDKO 20.81 ± 4.46%, *p* = 0.0214, two-way ANOVA with Bonferroni’s post hoc comparisons). **c** *Left:* Increased GFAP levels in the neocortex of IN-*PS* cDKO mice. Western blotting of total protein lysates from the neocortex of IN-*PS* cDKO mice and littermate controls at 3 and 9 months of age. β-actin was used internal loading control. *Right*: Quantitative analysis shows significant age-dependent increases of GFAP levels in the neocortex (F_1, 28_ = 0.41, *p* = 0.52; 3 M: *p* = 0.36, 9 M: *p* = 0.0341, two-way ANOVA with Bonferroni’s post hoc comparisons) of IN-*PS* cDKO relative to controls. The value of GFAP in littermate controls is set as 100%. **d** *Left:* Increased GFAP levels in the hippocampus of IN-*PS* cDKO mice. Western blotting of total protein lysates from the dissected hippocampus of IN-*PS* cDKO mice and littermate controls at 3 and 9 months of age. β-actin was used as internal loading control. *Right*: Quantitative analysis shows significant age-dependent increases of GFAP levels in the hippocampus (F_1, 28_ = 1.30, *p* = 0.26; 3 M: *p* = 0.53, 9 M: *p* = 0.0108, two-way ANOVA with Bonferroni’s post hoc comparisons) of IN-*PS* cDKO relative to controls. The value of GFAP in littermate controls is set as 100%. Scale bar: 100 μm. All data represent mean ± SEM. *p < 0.05. The value in the column indicates the number of mice used in each experiment

Since gliosis often accompanies ongoing neurodegeneration [11, 13, 14, 17, 41, 42], we further evaluated whether progressive gliosis is present in the cerebral cortex of IN-*PS* cDKO mice. We first performed immunohistochemical analysis of GFAP, a marker of reactive astrocytes, in IN-*PS* cDKO and control brains at the ages of 3 and 9 months (Fig. 8a, b). Stereological quantification of GFAP immunoreactivity showed significant increases in the neocortex (Control: 2.07 ± 0.22%, cDKO: 5.93 ± 1.39%, *p* = 0.0399, two-way ANOVA with Bonferroni’s post hoc multiple comparisons; Fig. 8a) and the hippocampus (Control: 6.57 ± 0.69%, cDKO: 20.81 ± 4.46%, *p* = 0.0214; Fig. 8b) of IN-*PS* cDKO mice at the age of 9 months. We also performed Western analysis using protein lysates from the dissected neocortex and hippocampus of IN-*PS* cDKO and controls at the ages of 3 and 9 months. Consistent with immunohistochemical results, we found significant increases of GFAP levels in the neocortex (*p* = 0.0341, two-way ANOVA with Bonferroni’s post hoc multiple comparisons; Fig. 8c) and the hippocampus (*p* = 0.0108; Fig. 8d) of IN-*PS* cDKO mice at 9 months of age.

**Fig. 9:**
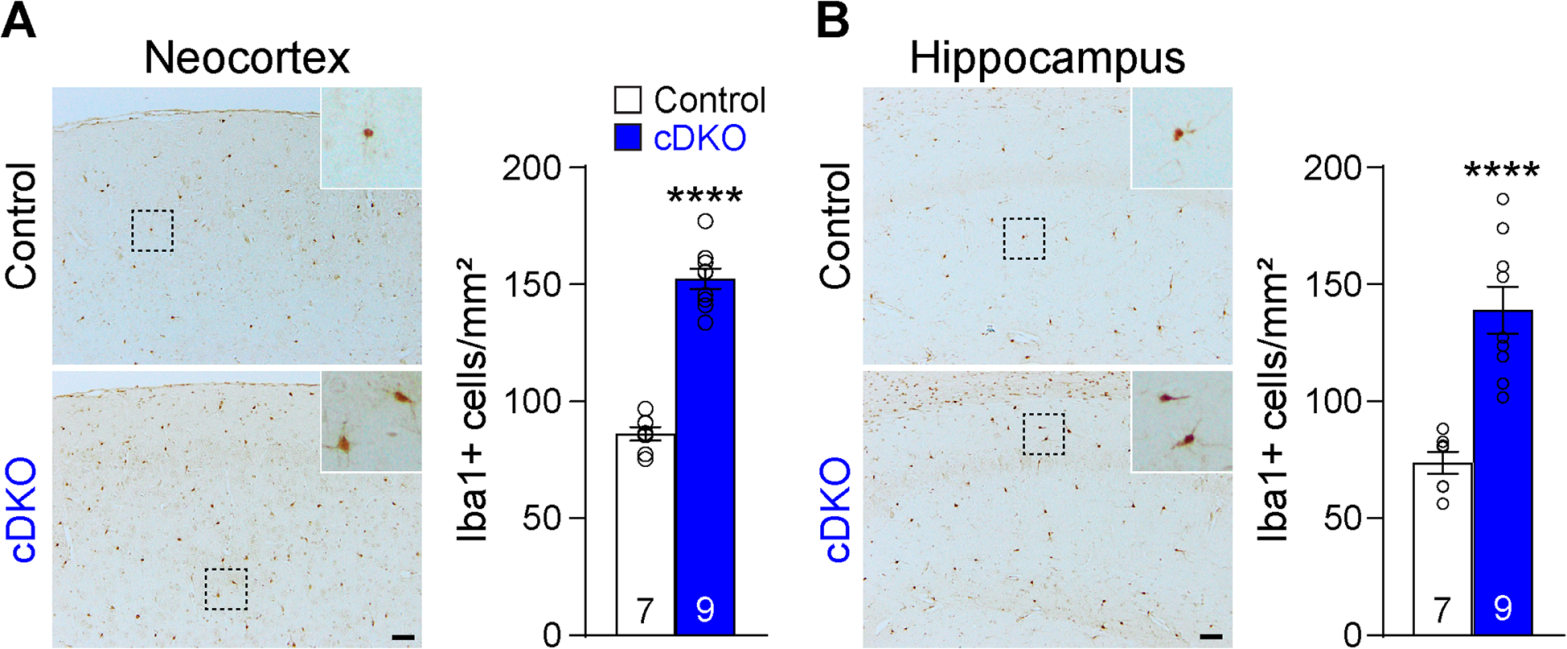
Microgliosis in the cerebral cortex of IN-*PS* cDKO mice. **a** *Left*: Iba1 immunostaining of comparable sagittal sections in the neocortex of IN-*PS* cDKO and littermate control brains at 9 months of age. Inserts show higher power views of the boxed areas. *Right*: Stereological quantification shows significant age-dependent increases in the number of Iba1-immunoreactive cells in the neocortex of IN-*PS* cDKO mice relative to control mice (Control: 86.17 ± 2.90 cells/mm^2^, cDKO: 152.40 ± 4.34 cells/mm^2^, *p* < 0.0001, Student’s *t*-test). **b** *Left*: Iba1 immunostaining of comparable sagittal sections in the hippocampus of IN-*PS* cDKO and littermate control brains at 9 months of age. Inserts show higher power views of the boxed areas. *Right*: Stereological quantification shows significant age-dependent increases in the number of Iba1-immunoreactive cells in the neocortex of IN-*PS* cDKO mice relative to control mice (Control: 73.70 ± 4.64 cells/mm^2^, cDKO: 139.00 ± 9.97 cells/mm^2^, *p* < 0.0001, Student’s *t*-test). Scale bar: 100 μm. All data represent mean ± SEM. **** *p* < 0.0001. The value in the column indicates the number of mice used in each experiment

We further evaluated microgliosis by performing immunohistochemical analysis using the microglial marker, Iba1, and saw increased Iba1 immunoreactivity in IN-*PS* cDKO brains at the age of 9 months. Stereological quantification showed significant increases of Iba1+ cells (Iba1+ cell number/tissue area) in the neocortex (Control: 86.17 ± 2.90 cells/mm^2^, cDKO: 152.40 ± 4.34 cells/mm^2^, *p* < 0.0001, Student’s *t*-test; Fig. 9a) and the hippocampus (Control: 73.70 ± 4.64 cells/mm^2^, cDKO: 139.00 ± 9.97 cells/mm^2^, *p* < 0.0001, Student’s *t*-test; Fig. 9b) of IN-*PS* cDKO mice compared to littermate controls. These data demonstrate that age-dependent loss of cortical interneurons in IN-*PS* cDKO brains is accompanied with astrogliosis and microgliosis.

## Discussion

More than 380 mutations have been reported in the *PSEN1* (320) and *PSEN2* (61) genes (https://www.alzforum.org/mutations), highlighting the importance of PS in AD pathogenesis. Previous genetic studies demonstrated that selective inactivation of PS in excitatory neurons of the cerebral cortex results in age-dependent, progressive neurodegeneration including increases of apoptosis and gliosis [11–13, 16]. Subsequent studies further showed that partial reduction of PS expression in excitatory neurons of the mouse cerebral cortex or in neurons of the *Drosophila* brain also causes age-dependent neurodegeneration and increases of apoptosis [17, 43]. In this study, we continue this reductionist approach to dissect the role of PS in inhibitory interneurons of the cerebral cortex through the generation and analysis of IN-*PS* cDKO mice, in which PS expression is selectively inactivated in inhibitory interneurons using *GAD2-IRES-Cre*, which was shown previously to drive Cre-mediated recombination in > 90% cortical interneurons [34].

Western analysis confirmed the reduction of PS1 in the neocortex (~ 15%) and hippocampus (~ 11%) of IN-*PS* cDKO brains (Fig. 1), consistent with the percentages of interneurons in the neocortex (15–20%) and hippocampus (10–15%), respectively [44]. The remaining PS1 detected on the Western blot is due to normal levels of PS1 expressed in excitatory neurons and glial populations, which lack Cre expression. Thus, the IN-*PS* cDKO brain is a mosaic, composed of interneurons lacking PS and excitatory neurons and glia expressing normal levels of PS1. The lack of PS in interneurons results in complete loss of γ-secretase activity, leading to dramatic accumulation of the physiological substrates APP CTFs in IN-*PS* cDKO brains (Fig. 1).

While IN-*PS* cDKO mice at 2 months of age exhibit normal spontaneous activities in the open field (Fig. 1), they begin to die at this age and their median lifespan is ~ 3 months. The exact cause of the early lethality in IN-*PS* cDKO mice is unclear. Given that interneurons in the brainstem and spinal cord are thought to control vital functions like breathing and swallowing, etc. [45–47], we speculate that interneuron dysfunction and degeneration caused by PS inactivation may underlie early lethality in IN-*PS* cDKO mice. Indeed, it was previously demonstrated that selective inactivation of PS in excitatory neurons results in synaptic dysfunction, such as impairments in neurotransmitter release, short-term and long-term synaptic plasticity, and intracellular Ca^2+^ homeostasis, followed by progressive, striking neurodegeneration [13, 15–18, 48, 49]. It therefore would be of great interest to perform similar electrophysiological analysis to determine the consequences of selective PS inactivation in interneurons on inhibitory and excitatory synaptic responses in the hippocampal local network and to investigate whether loss of PS in interneurons similarly disrupts intracellular Ca^2+^ homeostasis in a cell-autonomous manner.

Cre expression in IN-*PS* cDKO brains is under the control of the endogenous *GAD2* promoter, beginning approximately at embryonic day 10.5 [37]. Our quantitative histological analysis showed normal morphology and number of inhibitory interneurons in the cerebral cortex of IN-*PS* cDKO mice at 2 months of age, indicating normal generation and maturation of cortical inhibitory neurons in IN-*PS* cDKO mice (Fig. 2). However, as IN-*PS* cDKO mice age, they develop age-dependent loss of GAD67+ cortical interneurons as well as the PV+ and SST+ subgroups of interneurons in the cerebral cortex (Figs. 3, 4). The significant reduction of cortical interneurons in IN-*PS* cDKO brains at 9 months is preceded by increases of apoptosis at 3 months (Fig. 6). Furthermore, the increased apoptotic cells in the cerebral cortex of IN-*PS* cDKO mice are interneurons instead of excitatory neurons (Fig. 7). Thus, the significant reduction of cortical interneurons in IN-*PS* cDKO mice at 9 months is likely due to cumulative loss of apoptotic interneurons between 3 and 9 months of age. However, cortical volume (Fig. 3) and synaptic and dendritic markers (Fig. 5) are not significantly reduced in IN-*PS* cDKO mice at 9 months, likely due to the small percentage of interneurons in the cerebral cortex (10–20%), in contrast to ~ 35% reduction of cortical volume in EX-*PS* cDKO mice at 9 months of age because of > 80% excitatory neurons in the cerebral cortex [13, 16]. Furthermore, despite the small reduction of cortical interneurons (e.g. 22–24% PV+ and SST+ subgroups) in IN-*PS* cDKO mice at 9 months and the small percentage of interneurons in the cerebral cortex (10–20%), loss of cortical interneurons, which represents < 4% of all neurons, is accompanied with astrogliosis and microgliosis (Figs. 8, 9), similarly as previously reported in mouse models that exhibit age-dependent neuronal loss [11, 13, 16, 17, 50, 51].

Neuropathological changes in AD appear to occur first in the entorhinal cortex and hippocampus, and then extend to the rest of the neocortex [52]. Although most studies focused on the degeneration of excitatory neurons in the entorhinal cortex and hippocampus, vulnerability of interneurons has been reported in postmortem AD brains [25–30]. Specifically, loss of PV- and SST-expressing interneurons has been reported in the cerebral cortex of postmortem AD brains [26, 27, 31, 53–57]. Thus, age-dependent loss of PV- and SST-expressing interneurons observed in the neocortex and hippocampus of IN-*PS* cDKO mice (Fig. 4) may be relevant to similar loss of cortical interneuron in postmortem AD brains.

The molecular mechanism by which PS supports the survival of excitatory neurons and interneurons in the cerebral cortex is unknown. Since Notch receptors and APP family members are physiological substrates of PS, we previously conducted genetic studies to determine whether inactivation of Notch1/2 or APP/APLP1/APLP2 similarly causes age-dependent cortical neurodegeneration using the same *Camk2a-Cre* transgenic line that was used to inactivate PS [10, 13]. Surprisingly, no neuronal loss was observed in the cerebral cortex of *Notch1/2* cDKO mice [58] and *APP/APLP1/APLP2* conditional triple knockout mice [59], demonstrating that PS mediated cortical neuronal survival is not regulated through the Notch or APP family. Given the fact at any given time only 0.1–0.2% of excitatory or inhibitory cortical neurons lacking PS undergo apoptosis, making it impossible to perform biochemical analysis to gain insight into the molecular mechanism, we have therefore developed *Drosophila* models [43] and are now looking for genes that modulate age-dependent neurodegeneration caused by partial loss of Psn, the *Drosophila* homolog of *Presenilin*.

## Conclusions

Our current study shows that selective inactivation of Presenilin in interneurons results in age-dependent loss of cortical interneurons and increases of apoptotic interneurons as well as astrogliosis and microgliosis in the cerebral cortex. These findings, together with earlier reports, demonstrate a universal, essential role of PS in the survival of both excitatory and inhibitory neurons during aging.

## Supplementary Information

**Additional file 1.**

## Data Availability

The datasets generated and analyzed during the current study are available from the corresponding author upon request.

## Abbreviations

AD: Alzheimer’s disease
APP: Amyloid precursor protein
CaMKIIα: Calcium/calmodulin-dependent protein kinase type II subunit alpha
Cre: Cre recombinase
CTF: C-terminal fragment
EX-*PS* cDKO: Excitatory neuron-specific PS conditional double knockout
FAD: Familial Alzheimer’s disease
GABA: γ-aminobutyric acid
GAD: Glutamate decarboxylase
GFAP: Glial fibrillary acidic protein
HP: Hippocampus
Iba1: Ionized calcium-binding adapter molecule 1
IN-*PS* cDKO: Inhibitory neuron-specific PS conditional double knockout
IRES: Internal ribosome entry site
KI: Knockin
MAP 2: Microtubule-associated protein 2
NCX: Neocortex
PGK: Phosphoglycerate kinase
PS: Presenilin
PSD95: Postsynaptic density protein 95
PV: Parvalbumin
SNAP25: Synaptosomal-associated protein, 25 kDa
SST: Somatostatin
SYP: Synaptophysin
TUNEL: Terminal deoxynucleotidyl transferase dUTP nick end labeling
5HT3aR: 5-hydroxytryptamin (serotonin) receptor 3A
