# Supplementary material for: Cell-autonomous role of Presenilin in age-dependent survival of cortical interneurons
Source: Mol Neurodegener. 2020 Dec 10;15:72. doi: 10.1186/s13024-020-00419-y (PMC7731773; doi:10.1186/s13024-020-00419-y)
Supplement: Supplementary file 1 — Additional file 1. [file 13024_2020_419_MOESM1_ESM.pdf]

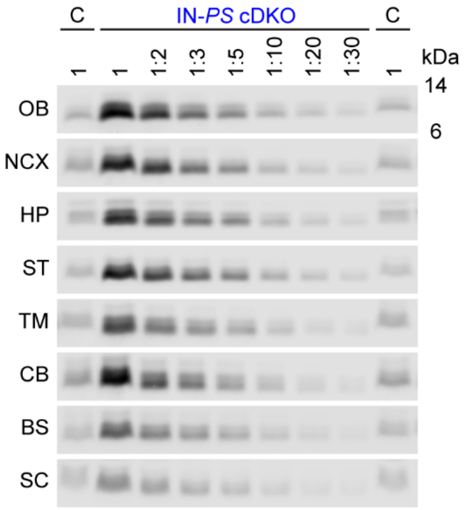

Supplementary Figure 1. Western analysis of APP CTFs using serial dilutions of brain lysates

Brain lysates prepared from the olfactory bulb (OB), neocortex (NCX), hippocampus (HP), striatum (ST), thalamus and midbrain (TM), cerebellum (CB), brain stem (BS), and spinal cord (SC) of IN-*PS* cDKO mice at the age of 2 months were diluted as indicated (1, 1:2, 1:3, 1:5, 1:10, 1:20, 1:30) and immunoblotted with the anti-APP-Y188 antibody along with the undiluted homogenate protein lysates from each brain sub-regions of littermate controls (marked as "C" in Lanes 1, 9).
